# A177 RISK OF VENOUS THROMBOEMBOLISM IN COVID-19 PATIENTS WITH INFLAMMATORY BOWEL DISEASE: A POPULATION-BASED MATCHED COHORT STUDY

**DOI:** 10.1093/jcag/gwac036.177

**Published:** 2023-03-07

**Authors:** R Khan, E Kuenzig, A Tang, J Im, J Widdifield, J McCurdy, G Kaplan, E Benchimol

**Affiliations:** 1 SickKids Inflammatory Bowel Disease Centre, Division of Gastroenterology, Hepatology and Nutrition, Department of Paediatrics, The Hospital for Sick Children, University of Toronto; 2 ICES; 3 Child Health Evaluative Sciences, SickKids Research Institute; 4 Institute of Health Policy, Management and Evaluation, University of Toronto; 5 Department of Medicine, University of Ottawa, Ottawa, Canada; 6 Department of Medicine and Community Health Sciences, University of Calgary, Toronto, Canada

## Abstract

**Background:**

Venous thromboembolism (VTE), is associated with significant morbidity and mortality. Inflammation increases the risk of VTE, and it is a well-recognised complication of both inflammatory bowel disease (IBD) and COVID-19.

**Purpose:**

To compare the risk of VTE among individuals with and without IBD following a positive COVID-19 test.

**Method:**

Using health administrative data from Ontario, Canada we conducted a retrospective matched cohort study.All Ontario residents with a positive SARS-CoV-2 PCR test between January 1,2020 and December 30,2021 who had been diagnosed with IBD prior to their COVID-19 infection (identified using a validated algorithm) were matched to 5 individuals without IBD based on year of birth, sex, mean neighbourhood income quintile, date of positive COVID-19 test, and rural/urban residence. Individuals with a cancer diagnosis in the 5 years prior to their first COVID-19 positive test were excluded. Individuals were followed from positive COVID-19 PCR test until VTE event, death, migration out of Ontario or March 31, 2022.VTEs were identified from emergency department or hospitalization data using ICD-10 codes. Incidence rate of VTEs among individuals with IBD were assessed at 1, 6 and 12 months. Proportional cause-specific hazards models compared the risk of VTEs in people with and without IBD, treating death as a competing risk and controlling for vaccination status (2^nd^ dose ≥14 days prior to positive COVID-19 test) and a history of VTE (VTE in the 5 years prior to infection).

**Result(s):**

There were 4293 people with IBD (44% Crohn’s disease, mean age ±SD 46.1±17.2 y) matched to 20,207 with out IBD (mean age 45.3±16.8 y) with a positive SARS-CoV-2 PCR test. Within 1 month of a positive COVID-19 test, the crude incidence rate of VTE in individuals with IBD was 4.77(95%CI, 4.75-4.80) per 100,000 person-days compared to 8.25(95%CI, 8.20-8.30) per 100,000 among people without IBD.Within 6 months, these rates were 1.86(95%CI, 1.86-1.87) and 2.12(95%CI, 2.11-2.12) per 100,000 person-days among people with and without IBD, respectivley. Within 12 months, these rates were 1.59(95% CI, 1.58-1.59) and 1.42(95% CI, 1.42-1.42) per 100,000 person-days among people with and without IBD, respectively.After adjusting for vaccination status and history of VTE there was no difference in the risk of VTE for people with and without IBD (HR 1.08, 95%CI, 0.64 to 1.83).

**Conclusion(s):**

IBD patients with COVID-19 were not more likely to experience a VTE infection compared with the general popluation. The risk of VTE was highest soon after COVID-19 and declined thereafter.

**Please acknowledge all funding agencies by checking the applicable boxes below:**

None

**Disclosure of Interest:**

None Declared

